# Insulin and exercise improved muscle function in rats with severe burns and hindlimb unloading

**DOI:** 10.14814/phy2.14158

**Published:** 2019-07-28

**Authors:** Juquan Song, Lisa A. Baer, Melody R. S. Threlkeld, Calvin Geng, Charles E. Wade, Steven E. Wolf

**Affiliations:** ^1^ Department of Surgery University of Texas Medical Branch and Shriners Hospitals for Children Galveston Texas; ^2^ Department of Surgery University of Texas Health Science Center at Houston Houston Texas; ^3^ Department of Surgery University of Texas Southwestern Medical Center Dallas Texas

**Keywords:** Genomic profile, isometric force, protein degradation, protein synthesis, signal regulation

## Abstract

Prior work established that exercise alleviates muscle function loss in a clinically relevant rodent model mimicking the clinical sequelae of severely burned patients. On the basis of these data, we posit that pharmacologic treatment with insulin combined with exercise further mitigates loss of muscle function following severe burn with immobilization. Twenty‐four Sprague–Dawley rats were assessed and trained to complete a climbing exercise. All rats followed a standardized protocol to mimic severe burn patients (40% total body surface area scald burn); all rats were immediately placed into a hindlimb unloading apparatus to simulate bedrest. The rats were then randomly assigned to four treatment groups: saline vehicle injection without exercise (VEH/NEX), insulin (5 U/kg) injection without exercise (INS/NEX), saline vehicle with daily exercise (VEH/EX), and insulin with daily exercise (INS/EX). The animals were assessed for 14 days following injury. The groups were compared for multiple variables. Isometric tetanic (Po) and twitch (Pt) forces were significantly elevated in the plantaris and soleus muscles of the INS/EX rats (*P* < 0.05). Genomic analysis revealed mechanistic causes with specific candidate changes. Molecular analysis of INS/EX rats revealed Akt phosphorylated by PDPK1 was increased with this treatment, and it further activated downstream signals mTOR, eEF2, and GSK3‐*β* (*P* < 0.05). Furthermore, muscle RING‐finger protein‐1 (MuRF‐1), an E3 ubiquitin ligase, was reduced in the INS/EX group (*P* < 0.05). Insulin and resistance exercise have a positive combined effect on the muscle function recovery in this clinically relevant rodent model of severe burn. Both treatments altered signaling pathways of increasing protein synthesis and decreasing protein degradation.

## Introduction

Muscle mass loss is dramatic following severe burn with subsequent function impairment (Clark et al., 1984). Loss of lean body mass and muscle atrophy are associated with delayed wound healing, increased infectious rate, and decreased physical and social quality of life (McClave et al., 1992). Furthermore, the impact of muscle loss is prolonged when patients are restricted and immobilized during hospitalization and recovery (Wolfe, 2001). Ultimately, immobilization complicates recovery from injury as much as the original injury itself. It has been observed that an early mobility program effectively decreases airway, pulmonary and vascular complications (Clark et al., 2013).

Current treatment is focused on stabilization of the injury condition. Comprehensive protocols include nutritional support, anabolic agents, physical therapy, and the prevention and treatment of sepsis and other complications (Rowan et al., 2015). Exercise has been proven to mitigate inflammation and the loss of muscle function in patients with other chronic diseases (Gielen et al., 2003) and during rehabilitation after burn (Diego et al., 2013). Suman et al. (2001) reported that resistance exercise training increased muscle mass and strength in pediatric burn patients during a 12‐week standard hospital rehabilitation program. Though protein degradation is considered the major mechanism of muscle mass loss after burn (Clark et al., 1984), exercise training could reset muscle protein turnover by increasing both synthesis and breakdown (Kumar et al., 2009). On a molecular level, exercise increases protein synthesis by activating the AKt‐mTOR‐p70S6k pathway (Wilkinson et al., 2008).

Insulin principally regulates glucose metabolism (Dimitriadis et al., 2011) and has been used as an intervention in burns, though its specific role remains in dispute (Demling, 2005). Insulin does increase protein synthesis by activating the PI3k/AKT pathway, which activates 4E‐BP‐1 and eEF2 to regulate initiation and elongation stages of protein synthesis (Proud, 2006). Insulin is also known to decrease protein degradation through the ubiquitin–proteasome system (Chen et al., 2011). However, insulin’s role in the AKt‐mTOR‐p70S6k pathway remains unknown.

The purpose of this study was to determine the impact of a combination therapy on muscle function recovery in a preclinical animal model for burn and hindlimb unloading. We posit that insulin and resistance exercise create an additive effect that improves the muscle function in burn and hindlimb unloaded (HLU) rats.

## Methods

### Animal procedure

**Figure 1:**
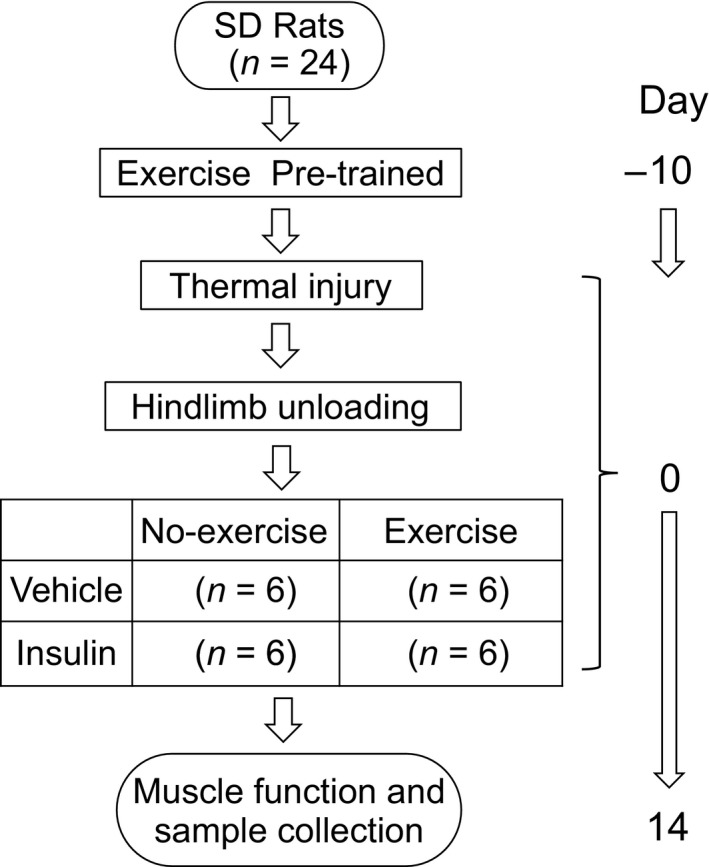
Study design flowchart ‐‐24 pretrained SD rats burn and hind limb unloaded at day 0. Animals were randomly assigned to four groups with insulin or exercise treatment for 14 days. Muscle force test in situ was performed following with sample collection.

Twenty‐four adult male Sprague–Dawley rats (Envigo, Houston, TX) were acclimated in specialized metabolic cages that were equipped with a traction system for HLU. Animals received exercise training 10 days prior to injury. During the training, the animals were assessed for the climbing ability and their resistance to participating in the task. All animals received a full‐thickness scald burn of 40% total body surface area and were placed in a harnesses and traction for HLU as previously described (Morey‐Holton and Globus, 2002). Animals were then randomly assigned for one of the following treatments: (1) saline vehicle without exercise (VEH/NEX), (2) insulin without exercise (INS/NEX), (3) vehicle with exercise (VEH/EX), and (4) insulin with exercise (INS/EX). An equal number of good and excellent climbers were included with each group. As appropriate, animal received 5 U/kg protamine zinc insulin (BCP Veterinary Pharmacy, Houston, Texas) daily subcutaneously, or 50 *µ*L saline (vehicle). The climbing exercise training procedure began the morning after injury and continued daily for 14 days. After 14 days, all animals were euthanized using standard of care protocols. The animal protocol was approved by the Institutional Animal Care and Use Committee (IACUC) at the University of Texas Health Science Center in Houston, followed the National Institutes of Health guidelines, and were described previously (Saeman et al., 2015). Animals were fed a powder diet (Harlan Teklad #2018) ad libitum and housed in a reversed 12‐h light/dark cycle during experiment. The detailed experiment is depicted in Figure 1.

### Exercise training

As previously described (Saeman et al., 2015), all animals underwent pretraining for 10 days before injury. Animals climbed a 1 m ladder covered in ½ inch plastic mesh at an 80° incline. Each session consisted of five climbs from bottom to top with a 10‐sec rest between repetitions. Pretraining consisted of 6 days of daily training sessions (am only) and then two sessions (am/pm) daily for the remaining 4 days. For this study, all rats successfully completed the exercise training protocol and were graded as either good or excellent. On the day of injury, rats were randomly assigned to groups. After injury, resistance weight was added with a harness, and was calculated daily as a percent of body mass of each rat and was gradually increased by 10% every few days as tolerated by the rats, with a maximum weight of 50% of body mass.

### Isometric muscle function testing

At 14 days after injury, animals were anesthetized with 2% isofluorane inhalation and the plantaris and soleus on the left hindlimb were isolated for in situ isometric muscle force measurement using the ASI muscle lever system with dynamic muscle control and analysis software (Aurora Scientific, Inc.) as previously described (Saeman et al., 2015). After reaching the muscle’s optimal length (Lo), isometric twitch and tetanic function were examined with a single 200 and 150 Hz electric stimulation with impulse duration of 0.2 msec at 10 mA in the plantaris and soleus consecutively. The fatigue function of the soleus was measured at 40 Hz with impulse duration of 0.2 msec at 10 mA for a total of 240 sec (Saeman et al., 2015).

### Histologic examination

Following the muscle function analysis, plantaris and soleus muscles were harvested from the left hindlimb and fixated in 10% neutral‐buffered formalin. Hematoxylin and eosin staining was performed on 5 *µ*m sections from paraffin‐embedded tissue. Quantitative analysis of soleus muscle fiber size was performed with blinded random cluster sampling of the muscle fiber cross‐sectional area. Image analysis was performed using Photoshop Adobe software.

### Total RNA extraction and genomic analysis

The plantaris tissue samples from the right limb of three random animals in each group were pooled, and the muscle tissue were processed at the microarray core facility in UT Southwestern Medical Center (Dallas, TX) for RNA extraction and gene expression measurements (Song et al., 2012). The Affymetrix rat gene ST 2.0 chip (Santa Clara, CA) was used for genome‐wide transcript expression change detection. Linear fold changes of signal data were analyzed with Affymetrix® Transcriptome Analysis Console (TAC 3.0) software. The interaction of target genes, gene ontology (GO) biological processing, and related pathways were also analyzed.

### Western blot detection

Thirty micrograms of protein was extracted from the right medial gastrocnemius muscle and analyzed by SDS‐PAGE and western blot following the previously described technique (Song et al., 2016). Band intensities were quantified with the Bio‐Rad ChemDom (Bio‐Rad Inc.). Glyceraldehyde‐3‐phosphate dehydrogenase (GAPDH) was utilized as loading controls. All antibodies including mTOR, Akt, eEF2, MuRF‐1, and GAPDH, etc. antibodies were purchased from Cell Signaling Tech (Danvers, MA) and Abcam (Cambridge, MA). SuperSignal West Pico and Femto Chemiluminescent Substrate were purchased from Thermo Scientific Inc. (Rockford, IL).

### Statistical analysis

Statistical analysis was performed with two‐way ANOVA followed by Holm–Bonferroni multiple comparison in Systat Sigma Plot®. Numbers reported as mean ± SEM.

## Results

### Muscle function

**Figure 2:**
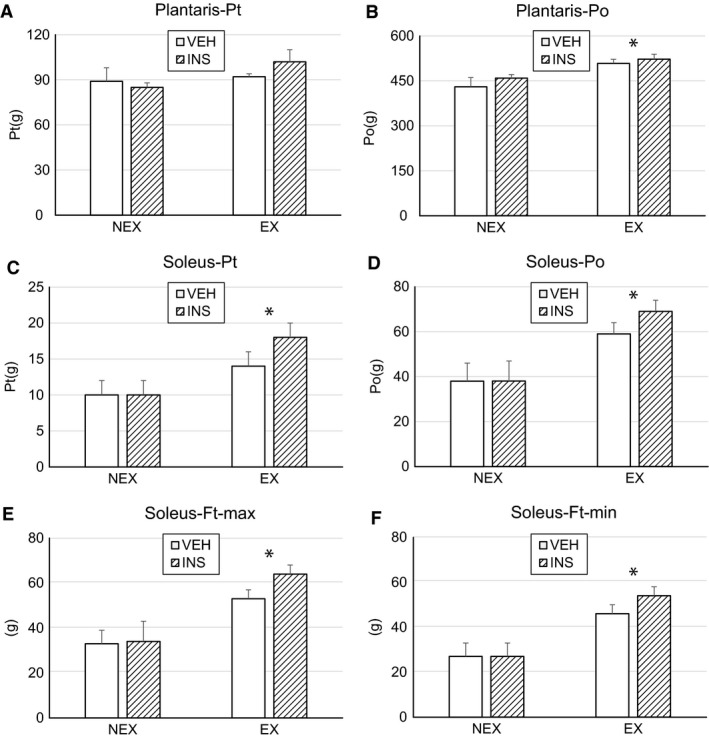
Muscle isometric force was measured in rat plantaris and soleus. *P < 0.05, exercise versus nonexercise, two‐way ANOVA.

Overall, exercise groups demonstrated improved muscle function when compared with those without exercise (Fig. 2). The soleus had significant elevation of Po (tetanic force), Pt (twitch force), fatigue maximum force, and fatigue minimum force in INS/EX rats compared to INS/NEX and VEH/NEX rats (Fig. 2). The significantly increased isometric force in both plantaris and soleus from INS/EX rats indicates that muscle function improved with insulin treatment in exercise‐trained rats.

**Figure 3:**
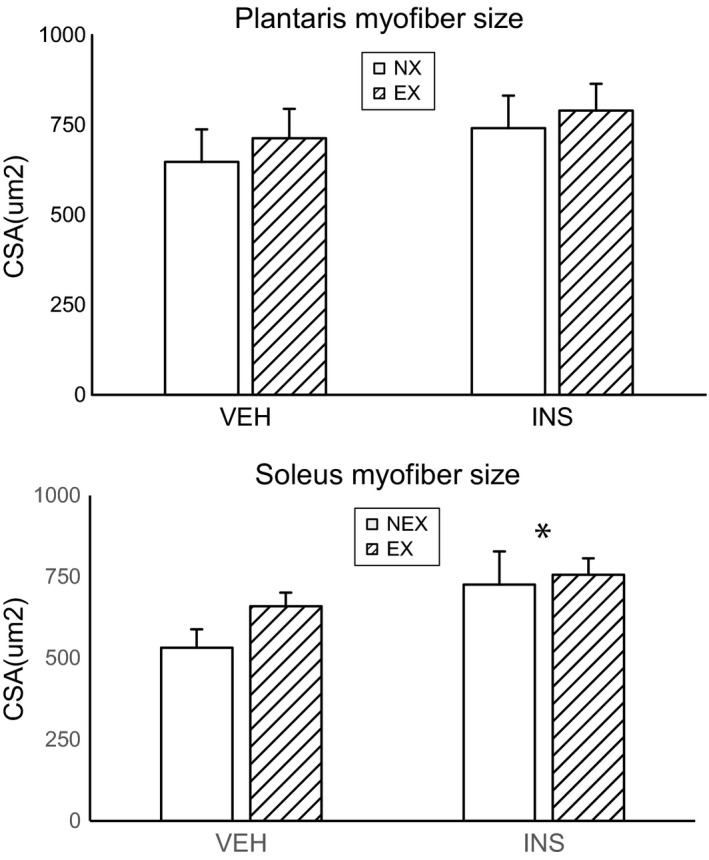
Muscle fiber size in plantaris and soleus. *P < 0.05, exercise versus non‐exercise, two‐way ANOVA.

Even though we found function changes, we did not detect a difference in either the plantaris or soleus muscle tissue mass between groups. Histologic estimation of muscle fiber size was significantly increased in the soleus with insulin treatment as compared to those treated with vehicle (Fig. 3). Plantaris muscle comparisons were not statistically different. Muscle physiological properties of both rat plantaris and soleus are reported in Table S1.

### Genetic and protein analysis

Genomic profile analysis showed that 122, 119, and 170 genes were affected (with an absolute fold change greater than 2) in INS/NEX, VEH/EX, and INS/EX, respectively. VEH/NEX was used as the comparison control. *Acta2* (*α*‐smooth muscle actin, regulating cell motility, structure and integrity), and *Sln* (sarcolipin, calcium channel regulator) were the most upregulated. *Mbp* (myelin basic protein, myelination of nerves), *Pmp2* (peripheral myelin protein 2, lipid transport), and *Mpz* (myelin protein zero, integral to membrane) were the most downregulated genes with insulin treatment and exercise (Table S2). Wikipathway analysis predicted that striated muscle contraction pathways were the most improved with in the INS/EX group, which directly correlates with the muscle function improvement. Meanwhile, TGF*β* and TLR signaling pathways were inhibited with both INS/NEX and VEH/EX treatments, indicating a decreased inflammatory response (Table S3).

PI3K/Akt pathway, the major intracellular signaling pathway that regulates cell cycle and protein synthesis through mTOR activation, was investigated. Protein kinase B (*Akt*) and 3‐phosphoinositide‐dependent protein kinase‐1 (*PDPK1*) gene expression were slightly upregulated in both INS/EX and INS/NEX groups (1.07 in INS/NEX, 1.05‐fold in INS/EX). *Akt1* increased 1.21‐fold, *Akt2* increased 1.1‐fold, and *PDPK1* increased 1.05‐fold in the INS/EX group (Fig. S1).

Proteasome‐dependent proteolysis pathway profiling revealed the highest number of downregulated genes in exercise groups, but not with insulin treatment (Fig. S2). The mRNA expression of E3 enzymes decreased in the INS/EX group, yielding an intensified net positive effect of combination treatment. Several genes showed varied expression when compared with the VEH/NEX control group. Most showed decreases of expression in INS/NEX, but increases in VEH/EX and INS/EX. These included the E3 ubiquitin–protein ligase, *Smurf‐1*; *TRIM63*, also known as *Murf‐1*; and *Fbxo32*, also known as *atrogin‐1*.

To investigate signal alterations in protein synthesis and degradation pathways at the protein level, we performed western blot analysis of the medial gastrocnemius muscle. Phosphorylated phosphoinositide‐dependent kinase 1 (PDPK1) activates Akt by phosphorylation of Akt at threonine 308, and Akt can be fully activated with phosphorylation at serine 473 as well. Western blot data confirmed that phosphorylated PDPK 1 and p‐Akt at T308/S473 expression increased when either insulin or exercise is used when compared with the VEH/NEX control (*P* < 0.05). Downstream signals, phosphor‐GSK3*β* and total mTOR, increased in all experimental groups as well. Elongation factor, eEF2, regulates protein synthesis and its expression significantly increased in the VEH/EX and INS/EX groups as compared to VEH/NEX (*P* < 0.05). The protein levels of muscle RING‐finger protein‐1 (MuRF‐1), a muscle E3 ubiquitin ligase for specific muscle protein degradation, was reduced significantly in the INS/EX group as compared to VEH/NEX (*P* < 0.05). The expression of atrogin‐1, another muscle specific E3 ubiquitin ligase, was not different between experimental groups and control (Fig. S3).

## Discussion

In our prior work, we observed that resistance exercise training added 50% body weight improved muscle function of the slow twitch dominated soleus in burn and hindlimb unloaded rats (Saeman et al., 2015). In this study, the positive effect of exercise on muscle recovery in burn subjects was once again confirmed. Specifically, exercise increased fatigue maximum force in the soleus of the experimental groups that included exercise regardless of whether insulin was used. Importantly, the combination of insulin treatment with exercise training had an additive effect on slow‐twitch myofiber dominate soleus muscle in burn and hindlimb unloaded rats. Increased single twitch force, maximum tetanic force and fatigue maximum force were all noted in the results. Moreover, exercise increased muscle isometric tension in the fast twitch myofiber dominated plantaris. This preclinical evidence suggests the advantage of combining insulin with exercise therapy in burn patients who are on bedrest.

Prior genomic profiling revealed that severe burns activate inflammation, metabolic glycolysis, gluconeogenesis, and ketone pathways; meanwhile disuse of the hindlimb muscles activate MAPK and oxidative stress pathways (Song et al., 2012). In addition, resistance exercise has been shown to downregulate oxidative and MAPK pathways while upregulating striate muscle contraction pathways (Song et al., 2012). The genetic profiling assays included herein supports the notion that exercise and insulin change the expression of the hypothesized and relevant signal pathways. These results aid in the identification of specific targets that regulate changes in muscle pathophysiology. Our analysis focused on two major pathways: PI3K/Akt/mTOR protein synthesis and ubiquitin protein degradation. p70S6k/eIF2 signals that regulate the initial stage of protein synthesis were not detected. It is possible that the pretraining period prior to experiment prevented the observation of these signals.

In contrast, the signals related to protein synthesis and protein degradation pathways were captured. Protein kinase B, Akt, is a major gate‐keeper of the protein synthesis pathway. Akt is regulated by phosphoinositide 3‐kinase (PI3K) and is associated with mTOR‐mediated protein synthesis. Insulin and other growth factors activate PI3K/PDPK‐1/Akt, which mediates survival signals and confers resistance to apoptosis. Resistance exercise activates the PI3‐AKT‐mTOR pathway increasing protein synthesis (Brooks and Myburgh, 2014). In this study, we found that PDPK1 increased phosphorylation of Akt in the INS/EX group, and Akt phosphorylated at both sites leads to protein synthesis. Another downstream action of Akt is GS3K‐*β* phosphorylation for glycogen synthesis, which was observed in the INS/EX group. The performance of Akt in this study indicates it is an important indicator of prognosis in burn patients.

Eukaryotic elongation factor 2 (eEF2) is a GTP‐binding protein that translates peptide elongation in protein synthesis, which is principally regulated by calcium‐dependent kinase eEF2K phosphorylation in burn patients (Song et al., 2012). In the genomic assays, *eEF2* expression was increased in all experimental groups (1.03‐fold in INS/EX, 1.01 in INS/NEX, 1.05 in VEH/EX). Gene expression changes were further confirmed with eEF2 protein expression using western blot detection. Both genomic and protein level examination indicates the protein synthesis increased through elevated eEF2 protein expression in burn/HLU rat muscle with insulin and exercise combination treatment.

The ubiquitin protein degradation pathway is one of the major pathways causing burn‐induced muscle atrophy. Muscle ring finger (MuRF‐1) and muscle atrophy F‐box (MAFbx/atrogin‐1) are two typical muscle specific E3 ligases in protein degradation. In 2006, Lang et al. reported that ubiquitin E3 protein atrogin‐1 and MuRF‐1 increased gene expressions by three and eightfold, respectively, 48 h after burn (Lang et al., 2007). With a fluctuant response afterward, MuRF‐1 mRNA was more transiently elevated and MAFbx/atrogin‐1 was persistently elevated 5 days after burn. They also found that MAFbx/atrogin‐1 and MuRF‐1 downregulated mRNA expression with IGF‐1 anabolic agent insulin‐like growth factor treatment. Compared with the baseline group (VEH/NEX), we found that the protein levels of MuRF‐1 significantly decreased in INS/EX rat muscle. Future evaluation of the time course of alterations in the signal pathways is warranted.

By further examining the muscle force and gene expressions in each group, we found that the effect of exercise is greater than insulin in this study. However, combination treatment has an even larger effect on muscle function when compared with individual therapy. There was no evidence that insulin alone increased muscle isometric force. Genomic profiling showed E3 ubiquitin ligase increased with insulin treatment, but it decreased with exercise training, which implies a mechanistic clue to the dominance of resistant exercise training in muscle function recovery in burn/HLU rats.

Severe burns cause insulin resistance and hyperglycemia (Jeschke et al., 2011), and insulin resistance is another molecular mechanism contributing to muscle atrophy (Wang and Pessin, 2013). Under pathophysiological stimulation, immature protein accumulates in the endoplasmic reticulum (ER) and activates the UPR pathway to desensitize insulin signals (Jeschke et al., 2011). Giving insulin with strict monitoring of the blood glucose level might conquer the barrier of insulin resistance and benefit patient metabolism without significant side effects (Jeschke et al., 2012; Pidcoke et al., 2014). In a previous study, insulin treatment decreased muscle loss in burn rats with hindlimb unloading, and activated IRS‐1/Akt pathway (Pidcoke et al., 2007). Similarly, we did not observe an apparent side effect of insulin, and animals were observed without behavior change during the experimental period.

Muscle function change is related to myofiber type, for instance fast‐twitch myofiber is less resistant to pathophysiologic stimulation during atrophy (Wang et al., 2006). Our previous studies indicate that muscle physiologic responses (Wu et al., 2010) and related mechanisms of satellite cells differ (Wu et al., 2013) between slow and fast twitch fibers after burn. We speculated that myofiber types are redistributed with insulin treatment or exercise training. However, we did not find the immunohistochemistry evidence of myofiber type changes in burn/HLU rat muscle using anti‐myosin type I and II antibody detection (data not shown).

In this study, rat muscle wet weight was not changed with either insulin or exercise treatment, yet muscle function improved. It is noted that the number of study subjects was small, which could limit the application of the results. However, both exercise and insulin have been shown to improve mitochondrial biogenesis and function via PGC‐1*α* (Pagel‐Langenickel et al., 2008; Safdar et al., 2011). The mechanism of “muscle quality improvement” should thus be further investigated.

Peripheral neuropathy creates a muscle function imbalance in differing groups of muscle, and should be considered when interpreting these data. Although the clinical observation of peripheral nerve complications after burn is not common (1982), Schaefer et. al reported a case of neurological injury after electrical burn (Schaefer et al., 2015). Less than 10% of burn patients could be found with clinical indications of motor and sensory abnormalities (Gabriel et al., 2009). However, genomic profile screening revealed possible damage to peripheral nerves following burn and HLU. Prior work from our lab confirmed that that *Mpz* expression increases 17.4‐fold, *Mbp* increases 8.55‐fold, *Pmp22* increases 6.4‐fold in the plantaris of burned rates with HLU at 14 days (Song et al., 2012), indicating myelination recovery in response to severe burn and muscle disuse. There is little information about muscle homeostasis related to peripheral nerve injury in response to severe burn. Martyn et. al studied that the number of muscle‐type acetylcholine receptors (AChR) increased after burn (Ward and Martyn, 1993), with elevated immature subunit transcript ACHRy (Ibebunjo and Martyn, 2001). The gene profile data might reflect peripheral nerve damage (such as demyelination). In this study, we found that insulin and exercise decreased myelination marker expression, implying increased recovery. Further investigation of severe burn related peripheral nerve injury is warranted for muscle function recovery as well.

### Perspectives and significance

Compared to the burn model without immobilization, the current animal model used in this study closely mimics a clinical scenario and provides a feasible platform to study the combination therapy of insulin and exercise. Although the effect of insulin alone was not shown, the additive effect of insulin with exercise was evident in both the plantaris and soleus of the injured rat. These data suggest muscle function improvement is involved with signal pathways regulating “muscle quality” rather than just purely muscle size/mass increase. Initial mechanistic studies were performed that are in line with prior literature. The outcomes presented herein are of clinical relevance and additional studies can delineate the specific mechanisms involved in recovery; these are sure to expand to additional systems including neurology, endocrinology, and cardiovascular circulation.

In conclusion, we found that insulin treatment has an addictive effect on the recovery of muscle function when resistance exercise therapy was used in an animal model of burn and HLU. The functional data were supported by exploratory gene expression assays. Further protein studies indicated that the combination of insulin and exercise alters the signal pathways associated with muscle function improvement. Moreover, we identified an increase in target signal proteins regulating protein synthesis and a decrease in protein degradation. By comparing the muscle pathophysiological changes with regulation mechanisms, we conclude that exercise has a greater effect than insulin on muscle function recovery.

## Conflict of Interest

The authors have no financial or other interests construed as a conflict of interest.

## Supporting information




**Figure S1A** **.** PI3K/Akt/NFκβ pathways in exercise.

**Figure S1B** **.** PI3K/Akt/NFκβ pathways in insulin.

**Figure S1C** **.** PI3K/Akt/NFκβ pathways in combined insulin and exercise treatment.

**Figure S2A** **.** Ubiquitin/proteasome protein degradation pathways in exercise.

**Figure S2B** **.** Ubiquitin/proteasome protein degradation pathways in insulin.

**Figure S2C** **.** Ubiquitin/proteasome protein degradation pathways in combined insulin and exercise treatment.

**Figure S3A** **.** Western blot images of signal protein expression in protein synthesis pathway including PDPK1, Akt, p‐Akt, mTOR,eEF2, and p‐GSK3β.

**Figure S3B** **.** Statistical analysis data of each protein expression in rat medial gastrocnemius. Two‐way ANOVA with post hoc Bonferroni test was applied +,INS/EX vs VEH/NEX; ', INS/NEX vs VEH/NEX; *, VEH/EX vs VEH/NEX; f, INS/EX vs INS/NEX; d, *P* < 0.05 INS/EX vs VEH/EX.

**Table S1** **.** Skeletal muscle function measurement in rat plantaris and soleus.

**Table S2** **.** Most altered genes with GO biological process (absolute value of fold change >2‐fold, ‐ out of range).

**Table S3** **. **Signal pathways with the number of altered genes (absolute value of fold change >2‐fold, ‐ out of range).
