# Supplementary figures and images for: Insulin and exercise improved muscle function in rats with severe burns and hindlimb unloading
Source: Physiol Rep. 2019 Jul 28;7(14):e14158. doi: 10.14814/phy2.14158 (PMC6661272; doi:10.14814/phy2.14158)

Exercise

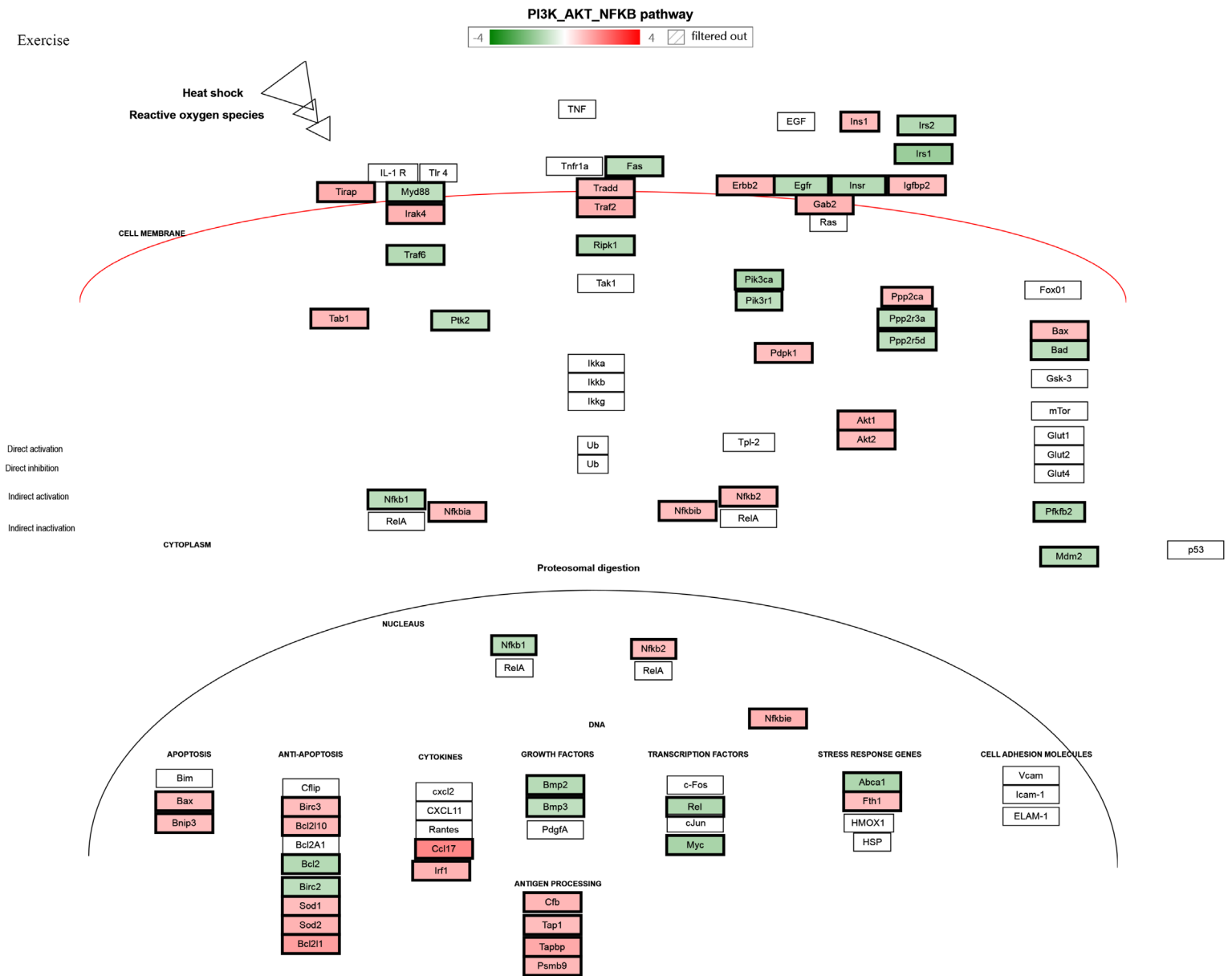

Supplemental Figure 1A

Supplement: Supplementary file 1 — Figure S1A . PI3K/Akt/NFκβ pathways in exercise. [file PHY2-7-e14158-s001.pdf]

Insulin

PI3K\_AKT\_NFKB pathway

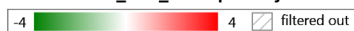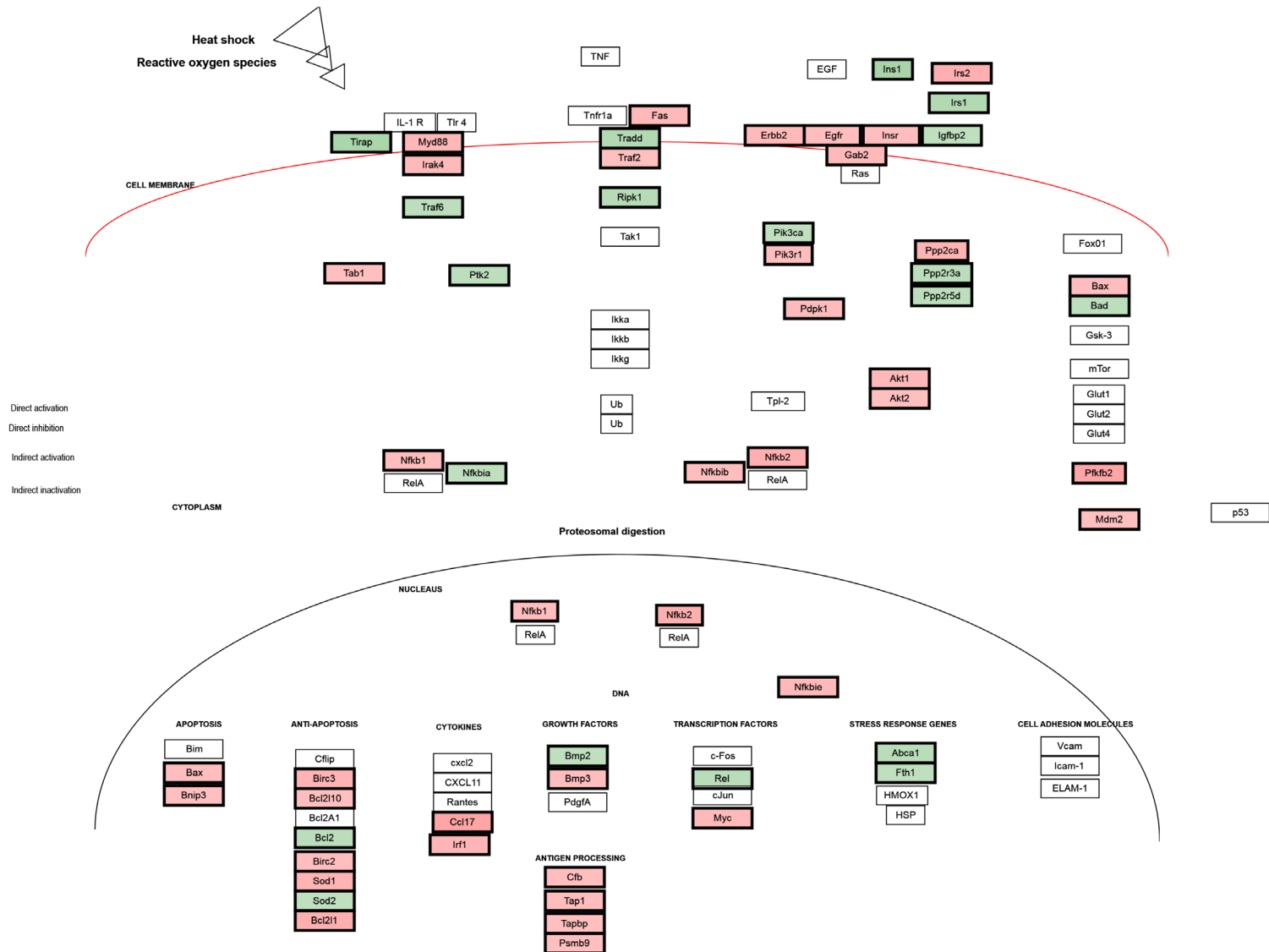

Supplemental Figure 1B

Supplement: Supplementary file 2 — Figure S1B . PI3K/Akt/NFκβ pathways in insulin. [file PHY2-7-e14158-s002.pdf]

Combination

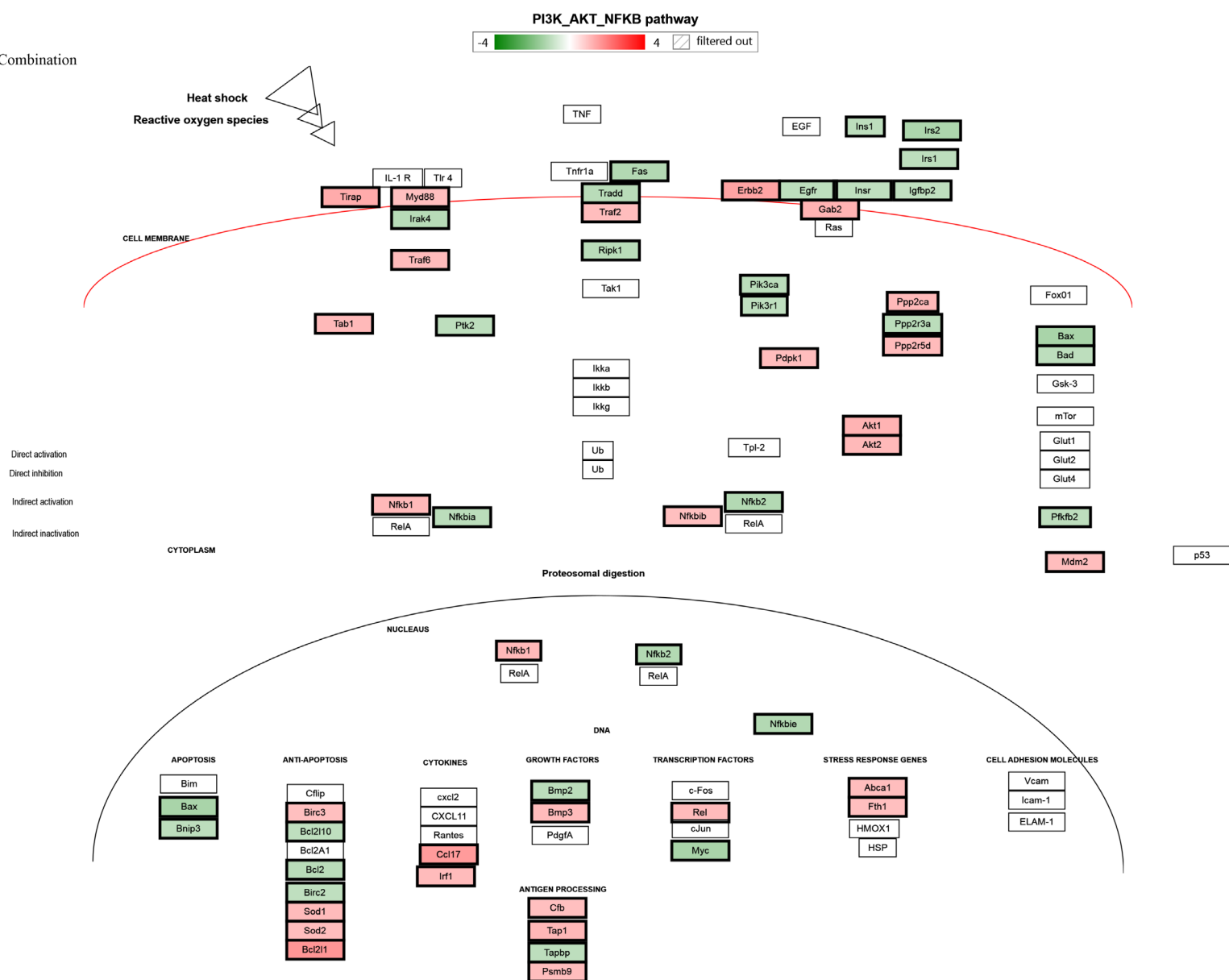

Supplemental Figure 1C

Supplement: Supplementary file 3 — Figure S1C . PI3K/Akt/NFκβ pathways in combined insulin and exercise treatment. [file PHY2-7-e14158-s003.pdf]

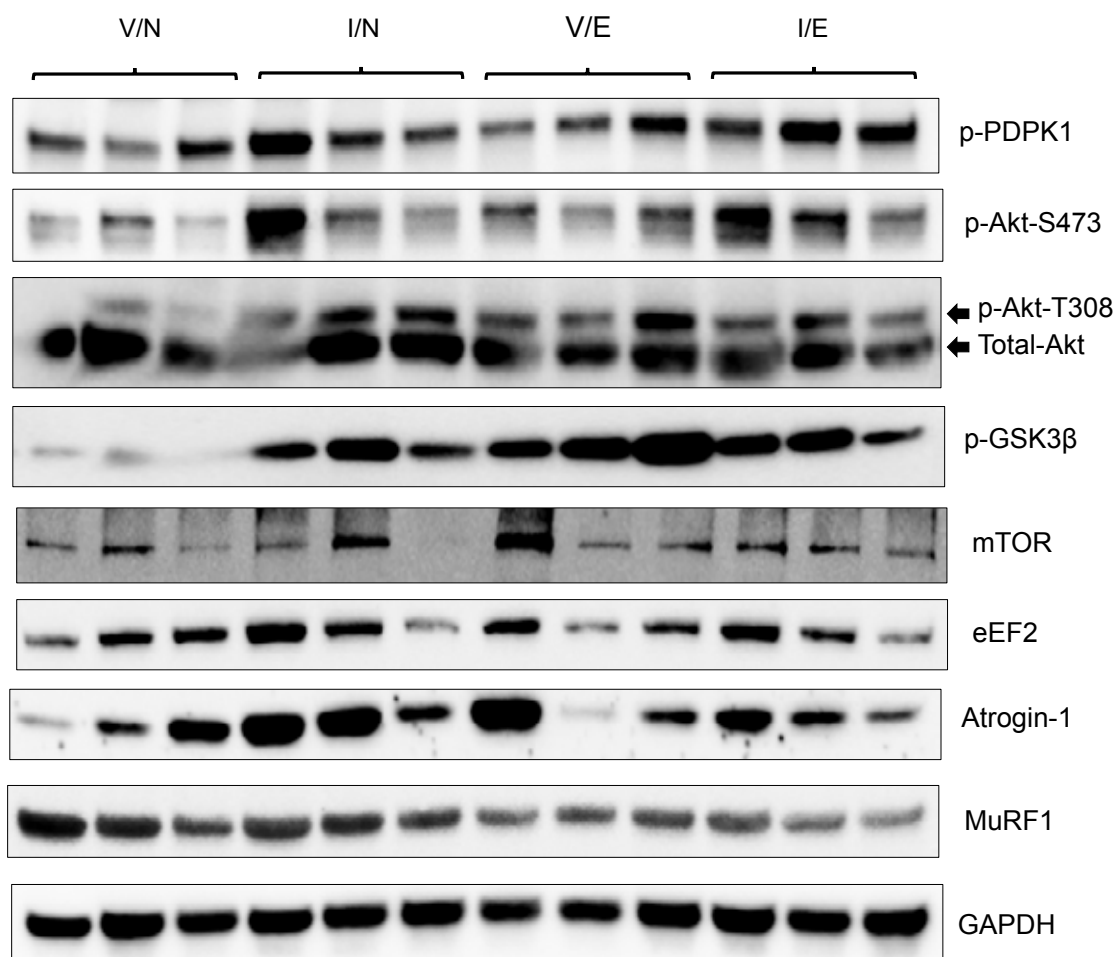

Supplemental Figure 3A

Supplement: Supplementary file 7 — Figure S3A . Western blot images of signal protein expression in protein synthesis pathway including PDPK1, Akt, p‐Akt, mTOR,eEF2, and p‐GSK3β. [file PHY2-7-e14158-s007.pdf]

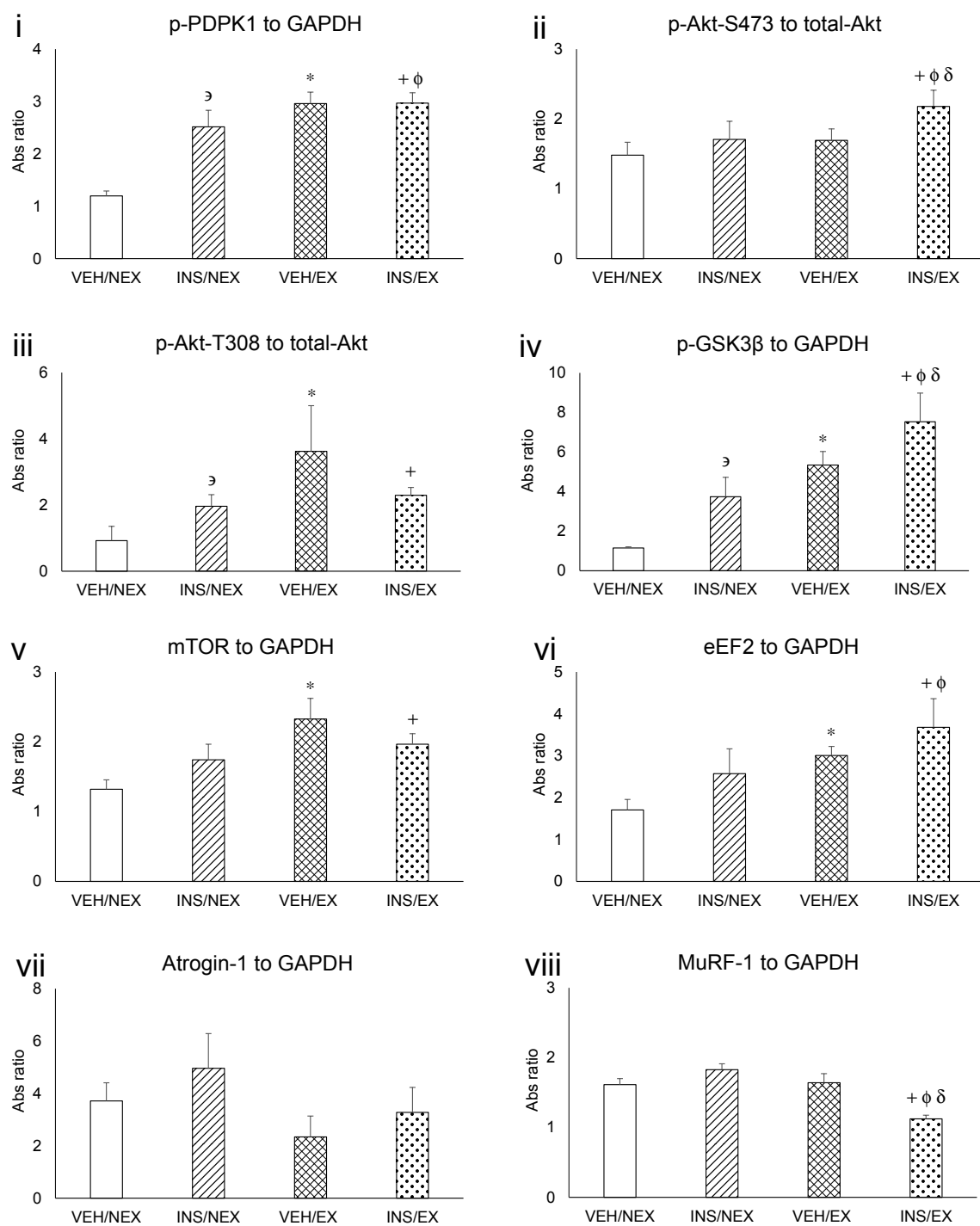

Supplemental Figure 3B

Supplement: Supplementary file 8 — Figure S3B . Statistical analysis data of each protein expression in rat medial gastrocnemius. Two‐way ANOVA with post hoc Bonferroni test was applied +,INS/EX vs VEH/NEX; ', INS/NEX vs VEH/NEX; *, VEH/EX vs VEH/NEX; f, INS/EX vs INS/NEX; d, P < 0.05 INS/EX vs VEH/EX. [file PHY2-7-e14158-s008.pdf]
