# Supplementary material for: Insulin and exercise improved muscle function in rats with severe burns and hindlimb unloading
Source: Physiol Rep. 2019 Jul 28;7(14):e14158. doi: 10.14814/phy2.14158 (PMC6661272; doi:10.14814/phy2.14158)
Supplement: Supplementary file 9 — Table S1 . Skeletal muscle function measurement in rat plantaris and soleus. [file PHY2-7-e14158-s009.docx]

**Supplemental Table 1.** Skeletal muscle function measurement in rat plantaris and soleus.

| Parameter |  | Group |  | Plantaris | | | |  | Soleus | | | |
| --- | --- | --- | --- | --- | --- | --- | --- | --- | --- | --- | --- | --- |
|  |  |  |  | No Exercise | | Exercise | |  | No Exercise | | Exercise | |
|  |  |  |  | Vehicle | Insulin | Vehicle | Insulin |  | Vehicle | Insulin | Vehicle | Insulin |
| Muscle |  | Wet weight (mg) |  | 332 ± 18 | 329 ± 9 | 348 ± 6 | 354 ± 14 |  | 119 ± 2 | 143 ± 23 | 151 ± 32 | 131 ± 7 |
|  |  | Lo (mm) |  | 35 ± 2 | 31 ± 0.3 | 32 ± 1 | 32 ± 1 |  | 33 ± 1.4 | 29 ± 0.4 | 31 ± 0.9 | 30 ± 0.4 |
|  |  | PCSA (mm^2^) |  | 27 ± 3 | 30 ± 2 | 30 ± 2 | 31 ± 2 |  | 5.0 ± 0.2 | 6.9 ± 2.3 | 6.7 ± 1.3 | 6.0 ± 0.3 |
| Twitch Force |  | Pt (g) |  | 89 ± 9 | 85 ± 3 | 92 ± 2 | 102 ± 8 |  | 10 ± 2 | 10 ± 2 | 14 ± 2 | 18 ± 1 ^*^ |
| Tetanic Force |  | Po (g) |  | 430 ± 31 | 459 ± 12 | 508 ± 14 | 522 ± 17 ^*^ |  | 38 ± 8 | 38 ± 9 | 59 ± 5 | 69 ± 5 ^*^ |
|  |  | Po/PCSA (N/cm^2^) |  | 16 ± 2 | 15 ± 2 | 16 ± 1 | 17 ± 1 |  | 7.4 ± 2 | 7.0 ± 1 | 10 ± 2 | 12 ± 1 |
|  |  | Pt/Po (%) |  | 21 ± 1 | 19 ± 1 | 18 ± 0.4 | 20 ± 1 |  | 26 ± 1 | 24 ± 2 | 24 ± 2 | 26 ± 2 |
| Fatigue |  | Maximum (g) |  | ----- | ----- | ----- | ----- |  | 33 ± 6 | 34 ± 9 | 53 ± 4 | 64 ± 4 ^*^ |
|  |  | Minimum (g) |  | ----- | ----- | ----- | ----- |  | 27 ± 6 | 27 ± 6 | 46 ± 4 ^†^ | 54 ± 4 ^*^ |
|  |  | Index (%) |  | ----- | ----- | ----- | ----- |  | 81 ± 7 | 84 ± 5 | 87 ± 5 | 84 ± 5 |

Lo = optimal muscle length

* EX vs. NEX (ANOVA, p<0.05)

PCSA = Physiological Cross Sectional Area

Pt/Po (%) = Ratio of twitch to tetanic force

Fatigue Index = Ratio of fatigue minimum to maximum
