# Supplementary material for: Insulin and exercise improved muscle function in rats with severe burns and hindlimb unloading
Source: Physiol Rep. 2019 Jul 28;7(14):e14158. doi: 10.14814/phy2.14158 (PMC6661272; doi:10.14814/phy2.14158)
Supplement: Supplementary file 10 — Table S2 . Most altered genes with GO biological process (absolute value of fold change >2‐fold, ‐ out of range). [file PHY2-7-e14158-s010.docx]

**Supplemental Table 2.** Most altered genes with GO biological process (absolute value of fold change >2-fold, - out of range).

| **Gene** | **Insulin** | **Exercise** | **Combination** | **GO Biological Process** |
| --- | --- | --- | --- | --- |
| *Actc1/2* | 3.38 | 5.38 | 3.88 | skeletal muscle thin filament assembly |
| *Sln* | 3.16 | 3.4 | 2.91 | calcium channel regulator activity |
| *Tecrl* | 2.72 | 2.13 | 2.18 | oxidoreductase activity |
| *Tnni1* | 2.38 | 2.19 | n/a | regulation of muscle contraction |
| *Tnnc1* | 2.45 | 2.25 | 2.08 | contractile fiber |
| *Mbp* | -3.03 | -4.02 | -3.44 | negative regulation of axonogenesis |
| *S100b* | -2.83 | -2.48 | -2.93 | receptor binding-zinc ion/s100/tau/CaDP/RAGE |
| *Scd* | -2.83 | -2.46 | -2.27 | fatty acid biosynthetic process |
| *Pmp22* | -3.67 | -2.98 | -3.25 | cell cycle arrest |
| *Pmp2* | -5.24 | -4.41 | -4.03 | fatty acid binding |
| *Mpz* | -15.14 | -10.07 | -8.52 | cell-cell junction maintenance |

Color formatted table red indicates gene upregulation and blue indicates gene downregulation.
