# Supplementary material for: Insulin and exercise improved muscle function in rats with severe burns and hindlimb unloading
Source: Physiol Rep. 2019 Jul 28;7(14):e14158. doi: 10.14814/phy2.14158 (PMC6661272; doi:10.14814/phy2.14158)
Supplement: Supplementary file 11 — Table S3 . Signal pathways with the number of altered genes (absolute value of fold change >2‐fold, ‐ out of range). [file PHY2-7-e14158-s011.docx]

**Supplemental Table 3.** Signal pathways with the number of altered genes (absolute value of fold change >2-fold, - out of range).

The length of bar stands for the number of altered genes (absolute liner fold change <2).
