# Supplementary material for: Insulin and exercise improved muscle function in rats with severe burns and hindlimb unloading
Source: Physiol Rep. 2019 Jul 28;7(14):e14158. doi: 10.14814/phy2.14158 (PMC6661272; doi:10.14814/phy2.14158)
Supplement: Supplementary file 6 — Figure S2C . Ubiquitin/proteasome protein degradation pathways in combined insulin and exercise treatment. [file PHY2-7-e14158-s006.pdf]

Combination

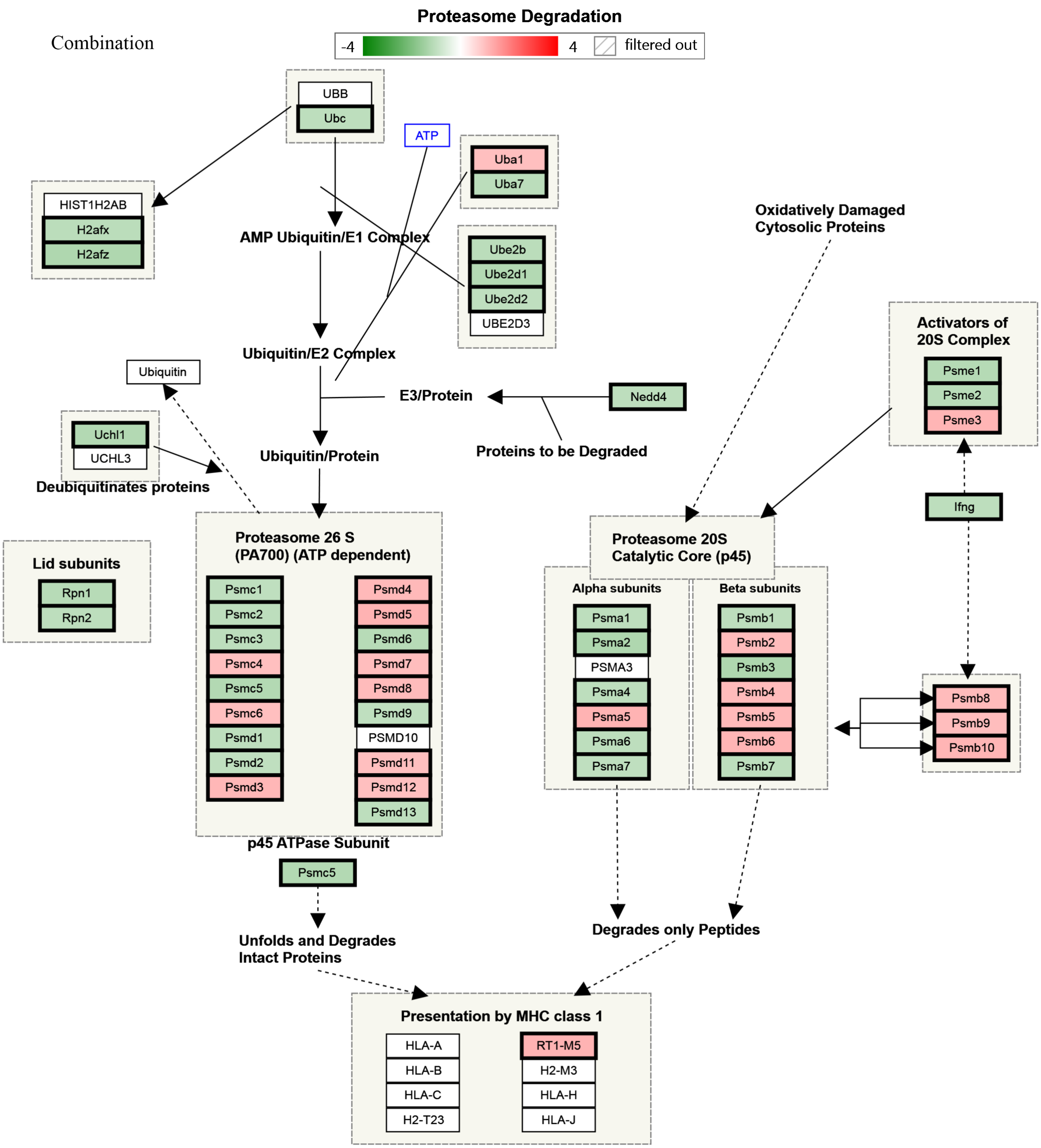

Supplemental Figure 2C

M8995
